# Home visit to premature and low birth weight newborns: nurse′s experience report

**DOI:** 10.1590/1980-220X-REEUSP-2023-0209en

**Published:** 2024-01-08

**Authors:** Ana Izaura Basso de Oliveira, Monika Wernet, Gabriele Petruccelli, Aline Oliveira Silveira, Mariana Torreglosa Ruiz

**Affiliations:** 1 Universidade Federal de São Carlos, São Carlos, SP, Brazil.; 2 Universidade de Brasília, Brasília, DF, Brazil.; 3 Universidade Federal do Triângulo Mineiro, Uberaba, MG, Brazil.

**Keywords:** Neonatal Nursing, Mothers, Infant, Premature, Infant, Low Birth Weight, Home visit, Poder Familiar, Enfermería Neonatal, Madres, Recién nacido prematuro, Recién Nacido de Bajo Peso, Visita Domiciliaria, Responsabilidad Parental, Enfermagem Neonatal, Mães, Recém-Nascido Prematuro, Recém-Nascido de Baixo Peso, Visita Domiciliar, Parentalidade

## Abstract

**Objective:**

To report the structures of the experience of nurse’s home visits to premature and low birth weight newborns.

**Method:**

This is a descriptive study of the experience report type, structured on the experience of the nurse authors in the development of 48 home visits in a city in the state of São Paulo and its microregion between August 2020 and 2021 with eight mothers of premature and low weight newborns.

**Results:**

The guiding documents “Home visit for families with preterm and low birth weight newborns” and “Strategy of guiding questions for home visits” were created and used to promote open narratives from parental caregivers about caring for at-risk newborns, creating a relational space aimed at joint construction.

**Conclusion:**

The documents used have favored home visits, helping nurses to establish professional bonds and build relational space through dialogue when conducting their activities in the home environment.

## RESUMO

**Objetivo:**

Relatar os estruturantes da experiência de visitação domiciliar realizada por enfermeiros aos recém-nascidos prematuros e de baixo peso.

**Método:**

Trata-se de estudo descritivo do tipo relato de experiência, estruturado na vivência das enfermeiras autoras no desenvolvimento de 48 visitas domiciliares, em cidade do interior paulista e sua microrregião, entre agosto de 2020 e de 2021, com oito mães de recém-nascidos prematuros e de baixo peso.

**Resultados:**

Foram criados e utilizados os documentos orientadores *“Visita domiciliar para famílias com RN Prematuro e de Baixo Peso ao Nascer”* e *“Estratégia de perguntas norteadoras para visita domiciliar”* para promover narrativas abertas dos cuidadores parentais sobre o cuidado com o recém-nascido de risco, criando um espaço relacional direcionado à construção conjunta.

**Conclusão:**

Os documentos utilizados favoreceram a condução das visitas domiciliares, auxiliando o enfermeiro a estabelecer vínculo profissional e construir espaço relacional através do diálogo na condução de suas atividades em ambiente domiciliar.

## RESUMEN

**Objetivo:**

Informar los factores estructurantes de la experiencia de visitas domiciliarias de enfermeros a recién nacidos prematuros y de bajo peso.

**Método:**

Se trata de un estudio descriptivo de informe de experiencia, estructurado sobre la vivencia de las enfermeras autoras en el desarrollo de 48 visitas domiciliarias en una ciudad del interior de São Paulo y su microrregión, entre agosto de 2020 y 2021, entre ocho madres de recién nacidos prematuros y de bajo peso.

**Resultados:**

Se elaboraron y utilizaron los documentos orientadores *“Visita domiciliaria para familias con RN Prematuro y de Peso Bajo al Nacer”* y *“Estrategia de preguntas guía para visita domiciliaria”* con el fin de promover narraciones abiertas de los cuidadores parentales sobre el cuidado del recién nacido de riesgo, creando así, un espacio relacional con miras a la construcción conjunta.

**Conclusión:**

Los documentos utilizados favorecieron la realización de las visitas domiciliarias, ayudando al enfermero a establecer un vínculo profesional y a construir un espacio relacional a través del diálogo en la conducción de sus actividades en el entorno domiciliario.

## INTRODUCTION

Preterm birth, birth occurring before 37 gestational weeks, is often associated with low birth weight, birth weight less than 2,500 grams. Both conditions are directly related to neonatal morbidity and mortality^(1)^, a component of impact on infant mortality and repercussions on the child’s health^(1–3)^.

Brazil is among the countries with the highest number of premature births per year in the world, including recurrence of prematurity among multiparous women with increasing rates^(1,2)^. It is also noted that 70% of child deaths in Brazil occur in the neonatal period and are linked to prematurity, with around 30 million of these children falling ill during their first days of life^(1–3)^.

In the meantime, seeking quality health care in this context is urgent and one of the strategic components is the home visit (HV). It is recognized as a potential strategy to promote continued care for children at home as long as it is structured in comprehensive and collaborative efforts, based on educational, humanized and comprehensive care^(1,4–6)^.

Furthermore, in Brazil and around the world, in the context of maternal and child care, there is a growing number of Home Visitation Program initiatives^(4,5)^. With regard to the neonatal population, in the national territory, the measures adopted in the home environment began with the elaboration of the Humanized Care Standard for Low Weight Newborns – Kangaroo Care established by the Ministry of Health through Ordinance GM/MS No. 1,683 of July 12^th^, 2007^(7)^. A better understanding of the needs of the neonatal public, especially premature newborns (PTNB) and low birth weight newborns (LBW) has led national bodies to dedicate themselves to promote safe and quality perinatal care in which parents and family members are involved in care^(7)^.

As a public policy, this was the first step towards establishing home care, specifically for the PTNB and LBW population. This measure strengthened the use of safe and responsible hospital discharge, preparation of the family for return home and monitoring of these newborns at the Primary Care level^(7)^, with an understanding focused on the longitudinal interaction between the family and the health professional as a differentiated opportunity for health assessment and guarantee of supportive care, with chances of expanding autonomy and resilience, especially in parenting issues^(1,5,6)^.

In this context, nurses are highlighted due to their training aimed at family health care, possession of basic and expanded skills that, when systematized, provide quality monitoring and support for women, families and children in transitional moments experienced with the birth of a child^(1,6)^.

All moments of transition promote change, in this sense, it is essential to recognize and understand the effects and meanings that the individual identifies and that occur with changes in the states of being. Thus, nurses’ interventions at home are targeted and continuous actions that provide openness to knowledge of the moments experienced and are capable of promoting benefits to mental health, child and maternal well-being, encouragement of parenting, family relationships and favoring responses individual and collective positives^(1,6,8)^.

In view of the above and given the urgency of supporting pregnancy, birth and parenting in the context of premature and low birth weight births^(1,5)^, the objective was to report the structures of the experience of home visits by nurses to premature and low weight newborns.

## METHOD

### Study Design

This is a descriptive study of the experience report type, structured based on the experience of the nurse authors in developing home visits nested within a doctoral study.

### Place

The experience took place in a city in the center-east of the state of São Paulo, whose estimated population, in 2021, was 256,915 inhabitants and in cities belonging to its microregion^(1)^, with a total of 3,503 births in 2020, 362 of which were below of 2,500 grams of body weight, that is, 10.3% of births and 359 PT births, totaling 10.2% of all births^(1)^. The Kangaroo Method is not a focus on the care of PTNB and LBW newborns in the city.

### Population and Selection Criteria

The recruitment and invitation to participate in the study was aimed at mothers of PTNB and LBW and occurred after the birth of the child, while the woman was still hospitalized, in a municipal philanthropic maternity hospital, considering the following inclusion criteria: living in the city of interior of São Paulo listed for the study or being from the microregion referenced to it; being monitored by the Unified Health System (SUS); not experiencing clinical complications; your child should be a low birth weight newborn and borderline or moderately premature, with discharge scheduled to occur together with the child. The exclusion criteria were: declaration of abusive use of psychoactive substances; being homeless or sheltered; your child is a twin and/or has a congenital malformation diagnosed in the maternity ward.

### Setting

The study setting was based on home visits to mothers of premature newborns and/or low birth weight newborns and focused on expanding accountability, continuity and instrumentalization for home care through maternal participation and empowerment in the process of return home with a view to parental care, health education, use of the kangaroo position and identification of elements that enhance maternal self-efficacy, based on relevant scientific evidence.

### Data Collection

The experience described took place through home visits between August 2020 and August 2021. An invitation was made to thirty-one women, from which seventeen refused to participate under the following justifications: fear of receiving a home visit due to the pandemic caused by the Sars-CoV-2 virus, family interference, social noise, maintaining privacy, mother’s demands related to household chores and child care. Therefore, eight women took part in this program for home visits. Each received six visits over four months, each lasting an average of 120 minutes. The purpose of the home visit was to talk about what it was like to care for a child at risk at home, providing support for this care and encouraging the use of the kangaroo position. All HV were conducted by the first author of the study under the support of a second visitor, all co-authors of this report.

### Data Analysis and Processing

The results are presented based on the experience of visiting nurses in the home care environment for premature and/or low birth weight newborns, with reflections and actions guided by literature review^(1,9)^ and the experiences found in the process of conducting of the study. The contents of the home visits were recorded individually in field diaries and synthesized into a single record with organization and transcription. The conduct of the study was based on the Theoretical Framework of the Theory of Transitions by Afaf I. Meleis^(8)^ and followed the Consolidated Criteria for Qualitative Research Reports – COREQ^(10)^.

### Ethical Aspects

This experience report originated from activities developed in a research that sought, among its specific objectives, to propose a guiding document for nurses’ home visits to mothers of premature and/or low birth weight newborns after hospital discharge. This study received approval from the Human Research Ethics Committee in 2020 under numbers 4,108,812 and 4,138,360, complying with the precepts of Resolutions 466/12 and 510/16 of the National Health Council. The inclusion of participants in the study was by signing the Free and Informed Consent Form (TCLE).

## RESULTS

Given the intention of providing support to women who are mothers of children born PT and BP from VD, there was a need to structure them. The structuring began with the question “What needs are common to the population of women who are mothers of children born PT and BP with a view to caring for this child? What is the evidence related to their experiences of parenting?” To this end, the scientific literature was mapped on the elements that constitute and support home visits aimed at mothers of low birth weight and premature newborns, published in 2021^(9)^. The essentiality and influence of the approach used by nurses in HV was revealed when listening efforts and offering support for family reorganization and maternal empowerment based on their knowledge, beliefs and values are central and contribute to strengthening the bond with the professional.

Thus, women needed to have the opportunity to narrate, to expose themselves, to expose their real needs in a context of sensitive listening and directed towards dialogue. Therefore, recognizing the existence of social constructions regarding particular care in the face of the condition of prematurity and LBW needs to be based on dialogue, with attention to the meanings conveyed and their determinants. Furthermore, there was our understanding that the precepts of kangaroo care corresponded to the needs of PT and BP children, parents and families, which led us to strategically encourage the use of the kangaroo position.

**Figure 1:**
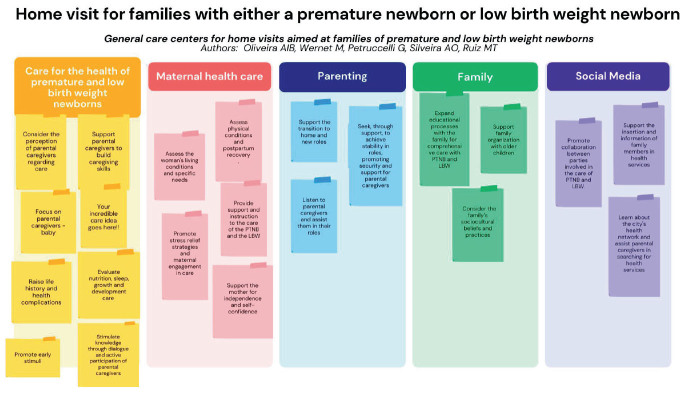
Home visit for families with premature and low birth weight newborns.

**Figure 2:**
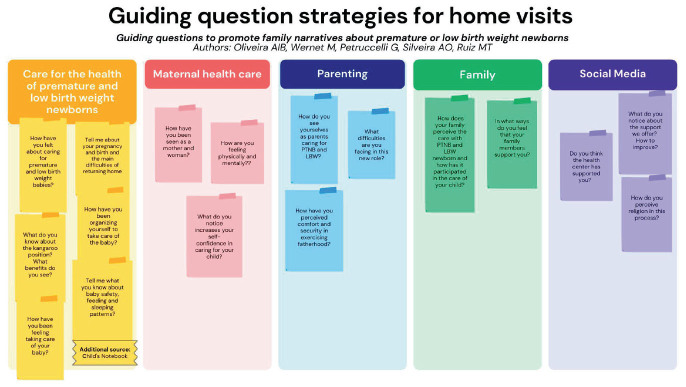
Guiding question strategy for home visits.

The approach to scientific evidence and collaborative dialogue between us culminated in the proposal of documents guiding HV for women with premature and low birth weight children, namely: “Home visits for families with premature and low birth weight babies” (Figure 1) and “Guiding question strategy for home visits” (Figure 2).

In front of these markers, the visiting team and its training were established. To this end, the proponent of the doctoral study invited people who were nurses and who, in her perception, shared the precepts presented above and were available to perform HV.

The group of visitors was made up of three people, the main doctoral researcher with specific lato sensu and stricto sensu professional training in the neonatal area, a nurse beginning stricto sensu training in the area of neonatology and a nursing graduate, at the time, with DV experiences are part of the literature mapping mentioned above.

The visitors carried out dialogic circles until they felt they were able to carry out the practical development of the HV. The conception was anchored in the clarity of the structures, both regarding knowledge and attitudinal aspects. In relation to the latter, the proposal was to focus on building a relational environment that promoted the narrative and the visitor would launch some nucleus based on the instruments, if the process did not spontaneously permeate it. Questions brought up and not present in the instruments were accepted, for example, questions regarding the pandemic caused by the Sars-CoV-2 virus, which brought great concerns and doubts during home visits.

HV began with the child’s return home. The first HV occurred as soon as possible for mother and baby, with the intention that its occurrence would occur within the first three days after discharge or at most in the first week after discharge, following documentary indications^(11,12)^. When monitoring a child born at risk, according to the Kangaroo Method (MC) guiding document, primary care (AB) is recommended to carry out three consultations in the first week, one of them at the HV level, two in the second week and a weekly appointment from the third week after the child’s hospital discharge until the child reaches a weight of 2,500 grams^(13)^. Prematurity and low birth weight constitute a profile of neonatal vulnerability with indications of continued care and close to PC upon hospital discharge^(14)^.

The visitors focused on approaching and creating a bond with the woman mother and her family, when the effort and commitment involved sensitive and empathetic listening towards understanding demands and needs, not only related to issues of child care, but also of walking in life as a whole.

The experience in an intermediate care unit made women who were mothers of premature and low birth weight babies try to learn as much as possible about how to care for their child, and it was from there that they gathered tools for returning home. Our meeting took place when their babies were not yet expected to be discharged, I established contact and, over time, we met in my search for the hospital care unit. The fact that we saw each other frequently made me understand that the professional-patient bond was established there. This way, they (mothers of premature and low birth weight babies) felt more confident in presenting their demands and finding me as support. [Field note from main visitor].

The understanding was that the totality reverberates in private experiences and vice versa, as well as the existence of complex crossings in this process. When listening, special attention was given to the externalization of feelings and, those close to suffering and hope, gained special attention.

It is clear that each phase is driven by a search. The beginning of returning home is maintained by the feeling of anxiety about the child’s well-being, nutrition and weight gain. As these concerns are resolved, new doubts and learning opportunities are necessary. [Field note].

## DISCUSSION

To create a bond between the nurse and the mother, we focused on encouraging narratives about their experience of motherhood with a premature child, resuming and embracing projections and what was experienced. The hospital discharge process after the birth of a premature and low birth weight child and their first months at home are seen as difficult moments with dual feelings of happiness and anguish. The birth and care of a premature and low birth weight child result in challenges arising from an unexpected way of caring and affect the mother’s quality of life, as well as her family and social life^(15)^. The dialogue unfolding from the narratives established in the HV allowed verbalization and accommodation of frustrations in the exercise and experience of the maternal role, as well as educational interventions related to the concepts that acted as restrictive to women’s autonomy. Actions guided and triggered in and by the context of life are powerful for identifying social determinants, beliefs and individual and social network resources and directing interventions for support, empowerment and autonomy. Home visits, when well established, favor the identification of vulnerable situations that expand in individual and collective circumstances^(16)^ as well as counterpoints to them.

In the first HVs, the maternal focus on the ‘correct’ performance of actions that meet the child’s basic human needs (especially hygiene, food and sleep) was notable. Thus, the conversations and questions were on this topic and, the fact that we were at home, favored narratives related to the inclusion of the partner/father in this process, with his clear desire to integrate him, despite the crossing of gender issues that end up referring women to the centrality of parental experiences. In this way, it is clear that gender issues permeate motherhood and dialogues aimed at countering them were busy topics in HV, especially after the perception of being able to meet the child’s needs. The woman feels exhausted by the demand and considers contacting people in her social network with reflections related to expanding the inclusion of the child’s father.

Recognizing and welcoming the partner to help the woman reduces maternal stress and anxiety, making intervention programs that significantly help support the mother^(17)^ crucial. The recognition of paternal importance given by the partner’s appreciation of activities with the child makes the father’s insertion in strengthening the parental bond positive and alleviates feelings of maternal anguish and stress. After discharge, the partner’s participation in caring for the child allows for fewer moments of insecurity for the mother, demonstrating how much this involvement has consequences for the baby’s life history and within the home environment^(18)^. However, for the above presence and participation, the insertion of the partner needs to occur from prenatal care and throughout labor and birth. It happens that he has always been placed on the margins^(19)^.

The woman mother weaves a process of reinterpretation of motherhood based on the redefinition of her conception of prematurity and low birth weight and the place of the father and the extended family, so that, for the visiting nurse, a support anchored in the mediation of these elaborations, unique to each woman. In this process, they experience moments of ‘crises’ and given the challenging circumstances for the personal-parental skills they are experiencing, they feel the need for continuous support and to talk more frequently with the visiting nurse, an aspect made possible by the adoption of an electronic instant messaging resource. Conversations established through messages provide greater flexibility in moments of exchange and create space to support new information and collaboration in moments of uncertainty, strengthening the construction of bonds and appreciation of assistance from the participants’ perspective^(20,21)^.

Therefore, it was identified that women needed greater proximity for involvement to be established, therefore, contact by electronic means was a resource used and was a supporting and facilitating tool in coping with daily difficulties^(21).^

Nursing practice through telephone communication or virtual social networks facilitates access to health, informational support and monitoring for mothers of premature babies. Furthermore, it improves hope and perceived maternal self-efficacy^(22)^. Increased hope is related to the ability of women mothers to reduce their stress and anxiety, as well as overcome despair with guidance and an appropriate approach^(22)^. Maternal beliefs in their skills and abilities are a vital positive psychological factor for coping and performing through life’s challenges and incidents, such as premature birth, with repercussions on the better quality of the mother-child relationship^(22)^.

It is up to the nurse to identify the maternal perspective regarding the care that restricts premature and low birth weight newborns and, from then on, enrich the maternal knowledge, skills and self-confidence of the woman to carry it out, with contributions to identity maternal. The nurse brings to the home meeting ideals and reflections that emerge individually from the relationship established with parental caregivers, as well as creating the intervention based on it in alignment with existing scientific evidence. The basis for the composition of care elements is immersed in the history of life and the processes experienced, as they guide which paths have already been taken and which are still unknown.

Within the elements of care and as an early stimulus is the kangaroo position, with positive scientific evidence for the mother and the newborn^(23)^ such as better thermal regulation and physiological stability of the child, promotion of breastfeeding (BF), with expansion of milk production and its duration, positive stimulus to neurobehavioral development and effects on pain conditions^(23,24,25,26)^.

The use of the kangaroo position at home was desirable in the construction of HVs, however we identified that the majority of mothers had little information about the position, few had prior knowledge about it. Few women spoke about it, demonstrating how the Kangaroo Mother Care has been approached in an incipient way in prenatal care, birth and follow-up of women who carry, give birth and care for a premature child with low birth weight.

Another point to be highlighted is maternal health, a pressing condition for better child care conditions. Women mothers tend, in initial meetings, to report little about themselves and focus almost exclusively on the health and well-being of their child, overriding care for themselves^(6)^. However, over time, they report tiredness and begin to talk about themselves, their exhaustion, their needs, with opportunities to promote reflections regarding maternal mental health.

Interaction with the child is often impaired due to maternal stress and anxiety with feelings of inability to care for the child, parental burnout, a term related to emotional exhaustion due to caring for the newborn and which can result in distancing from the mother-child binomial. It must be identified and treated early so that the relationship initiated is preserved and there is greater engagement with the care of PTNB and NBW^(27)^.

The physical conditions of postpartum recovery are little evidenced by maternal reports, despite being a phase of great physical, mental and emotional changes, the mother appears shyly alongside the risk birth, however, mothers of PTNB and LBW have greater chances of problems in relation to their physical and mental health, being more prone to postpartum depression than women who gave birth to full-term babies^(28)^. They often take this position, perhaps due to the insensitivity of health professionals to women, combined with interactions that reinforce the child’s centrality and the maternal duty to carry out care determined by the professional, sometimes immersed in judgments. This context inhibits the establishment of bonds and authentic relationships between women, family and professionals, as well as being restrictive to the process of maternal autonomy in child care. Throughout the HV, largely due to the dialogical proposal, a growing process of trust and authentic self-display in meetings was evident, favoring relational mutuality.

There is an urgent need for shifts in the practical exercise of HV, in particular, in replacing the reduced understanding of it being ‘the act of entering a home to obtain and give health information’ in the direction of investing in the establishment of a relational space in the home^(29)^ for the purposes to accommodate particular needs. The willingness for collaborative construction is the greatest challenge of HV as it is not safe to say that it is clear in its full meaning. The process of constructing respectful and dependent care for others is essential, driven by their particularity and not individuality^(30)^. Therefore, it presents a challenging task for the nurse when mobilizingzar the uniqueness of dialogue in the construction of new parental skills and with the recognition that transitional periods are surrounded by imbalances, uncertainties and personal and social conflicts^(9)^.

Adaptations to the home environment in response to the arrival of a premature and low-weight child place parental caregivers, especially the mother, in movements of perception regarding their way of understanding the new roles that are constructed from their cultural insertion and influenced by the over the years due to life contexts that internalize different ways of feeling and viewing oneself as competent and skilled in carrying out actions that are understood in parenthood.

Maternal self-efficacy is directly related to the support of parental caregivers in the care of premature and low birth weight babies and, although motherhood suggests that the woman has responsibilities for her child, in premature birth, her experiences and knowledge are devalued, being denied to them the authority and management of the care of the child, despite being responsible.

Finally, it should be noted that the quality of the home visit is closely linked to the time spent by the professional providing assistance and the intention designed for carrying it out. The average time for HV experienced in this study is about 120 minutes, a time considered opportune so that the researchers could enter the maternal universe and make efforts towards intersubjective care. This reality proves to be challenging in the Brazilian scenario, assuming the growing number of live births in the country, especially high-risk newborns who require home health care and the tiny number of professionals dedicated to such work^(14)^.

## CONCLUSION

The report defends the structuring of HV aimed at parental caregivers who are caring for children born preterm and with low birth weight, using a dialogical approach to encourage the exposure and acceptance of particularities. Furthermore, it was found that as the bond between visiting nurse and mother is established, prompt contact by electronic means for reception is a supporting factor to be required in current times.

The documents created and used favored the conduction of conversations in HV for this population followed by great weaknesses in the health services. The ‘being’ professional nurse favored knowledge for a ready technical dialogue and the clarity of being the horizon of care, sensitive listening and collaborative dialogical efforts, was fundamental for the establishment of a relational and care space.

In this context, the home support offered is associated, through maternal verbalization, with positive health practices and, even though there is a public health policy structure designed for this purpose and which uses home visits, they perceive it as a new action that is not included completely in the reality of the population, despite its theoretical structuring being widespread, which leads us to understand that care actions carried out at home need to be further worked on and implemented using tools that bring the professional closer and direct them to the particularities arising from the experience maternity care in situations of prematurity and low birth weight.
